# Ustiloxin G, a New Cyclopeptide Mycotoxin from Rice False Smut Balls

**DOI:** 10.3390/toxins9020054

**Published:** 2017-02-10

**Authors:** Xiaohan Wang, Jian Wang, Daowan Lai, Weixuan Wang, Jungui Dai, Ligang Zhou, Yang Liu

**Affiliations:** 1 Department of Plant Pathology, College of Plant Protection, China Agricultural University, Beijing 100193, China; wangxiaohan99@126.com (X.W.); wangjiancau@163.com (J.W.); dwlai@cau.edu.cn (D.L.); wwxcau@163.com (W.W.); 2 State Key Laboratory of Bioactive Substance and Function of Natural Medicines, Institute of Materia Medica, Chinese Academy of Medical Science & Peking Union Medical College, Beijing 100050, China; jgdai@imm.ac.cn; 3 Institute of Food Science and Technology, Chinese Academy of Agricultural Sciences, Beijing 100193, China

**Keywords:** ustiloxin G, cyclopeptide, mycotoxin, rice false smut disease, *Villosiclava virens*, rice false smut balls, cytotoxicity, phytotoxicity

## Abstract

Ustiloxins were cyclopeptide mycotoxins from rice false smut balls (FSBs) that formed in rice spikelets infected by the fungal pathogen *Ustilaginoidea virens*. To investigate the chemical diversity of these metabolites and their bioactivities, one new cyclopeptide, ustiloxin G (**1**), together with four known congeners—ustiloxins A (**2**), B (**3**), D (**4**), and F (**5**)—were isolated from water extract of rice FSBs. Their structures were elucidated by analyses of their physical and spectroscopic data, including ultraviolet spectrometry (UV), infrared spectroscopy (IR), 1D and 2D nuclear magnetic resonance (NMR), and high-resolution electrospray ionization-mass spectrometry (HR-ESI-MS). All the compounds were evaluated for their cytotoxic as well as radicle and germ elongation inhibitory activities. Ustiloxin B (**3**) showed the best activity against the cell line BGC-823 with an IC_50_ value of 1.03 µM, while ustiloxin G (**1**) showed moderate activity against the cell lines A549 and A375 with IC_50_ values of 36.5 µM and 22.5 µM, respectively. Ustiloxins A (**2**), B (**3**), and G (**1**) showed strong inhibition of radicle and germ elongation of rice seeds. When their concentrations were at 200 µg/mL, the inhibitory ratios of radicle and germ elongation were more than 90% and 50%, respectively, the same effect as that of positive control (glyphosate). They also induced abnormal swelling of the roots and germs of rice seedlings.

## 1. Introduction

Rice false smut is one of the most destructive rice fungal disease caused by ascomycete fungus *Villosiclava virens* Tanaka and Tanaka (anamorph: *Ustilaginoidea virens* Takahashi) [1]. It is a unique disease in the sense that symptoms are only visible after flowering, where the fungus infects individual spikelets and replaces the seed with a large orange to green ball, commonly known as false smut ball (FSB) [2]. The importance of this disease has risen recently, with the change of its status from “minor” to “major” causing concern in almost all rice cultivation regions across the globe [3]. In particular, this disease has been estimated to occur in one-third of the rice cultivation areas in China [4].

Mycotoxins are known as the toxic secondary metabolites produced by fungi. These toxic fungal metabolites are considered to number in the thousands, and are of concern for human and animal diseases. About 25% of food crops in the world are contaminated by mycotoxins each year, which leads to both considerable financial damage and safety risks [5]. Chemical investigations of the rice FSBs have led to the isolation and identification of two kinds of mycotoxins, namely ustiloxins and ustilaginoidins [6,7,8,9,10,11,12,13]. Ustiloxins belong to a group of water-soluble cyclopeptides containing a 13-membered cyclic core structure with an ether linkage [6,7]. They exhibit a variety of biological activities, including antimitotic activity by inhibiting microtubule assembly and skeleton formation of the eukaryotic cells, and have been regarded as potential antitumor agents for clinical applications [14,15]. When domestic animals were fed with the rice grains and feedstuff contaminated by the rice false smut pathogen, they showed a variety of symptoms such as diarrhea, hemorrhage, poor growth, ovarian atrophy, abortion, and damage of liver, heart, and kidney [10]. Furthermore, the crude water extract of rice FSBs was found to cause necrosis of the liver and kidney in mice quite similar to that observed in mice lupinosis caused by phomopsin A [16,17]. Meanwhile, ustiloxins also functioned as phytotoxins by inhibiting the radicle and plumule growth during seed germination of rice, wheat, and maize; inducing an abnormal swelling of the seedling roots; and resulting in the growth reduction and necrotic and dead frond tissue of duckweed (*Lemna pausicostata*) [15,18].

In our ongoing search for chemical diversity and biological activities of the ustiloxins, one new compound, namely ustiloxin G (**1**), along with four known ustiloxins (**2**–**5**) were isolated from the water extract of rice FSBs. The isolated ustiloxins were all evaluated for their cytotoxicity against human cancer cell lines, and phytotoxicity on the radicle and germ elongation of rice seeds. Herein, we described the isolation and structural elucidation of the ustiloxins from rice FSBs, as well as their biological activities.

## 2. Results and Discussion

**Figure 1:**
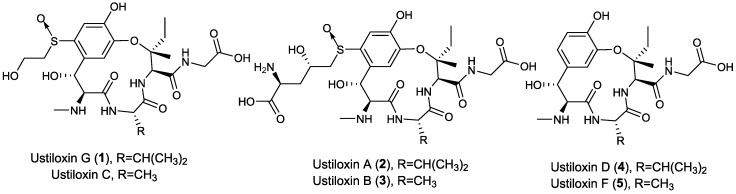
Structures of ustiloxins C, G (**1**), A (**2**), B (**3**), D (**4**), and F (**5**).

The water extract of rice FSBs was successively subjected to the repeated column chromatography over macroporous adsorption resin SP207, ODS-AQ, Sephadex G-15, and reversed-phase high-performance liquid chromatography (HPLC) to afford one new cyclopeptide **1**, along with four known cyclopeptides **2**–**5** (Figure 1). The known cyclopeptides were identified as ustiloxins A (**2**) [6,8], B (**3**) [6,8], D (**4**) (Figures S1 and S2) [6], and F (**5**) (Figures S3 and S4 in Supplementary Material) [7] by comparing their physical and spectroscopic data with those in the literature.

### 2.1. Structure Elucidation of Compound ***1***

**Figure 2:**
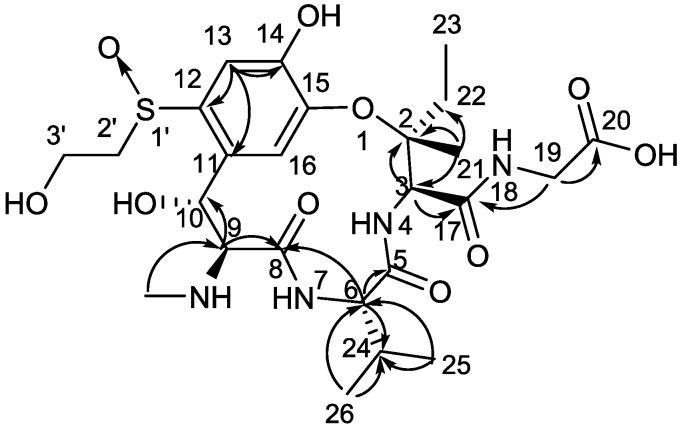
Key HMBC correlations (H → C) observed for ustiloxin G (**1**).

Compound **1** was isolated as a yellow amorphous solid. [*α*]${}_{D}^{24}$ −8.1 (*c* 0.2, H_2_O); UV (H_2_O) *λ*_max_ (log *ε*) 216 (3.83), 253 (3.50), 291 (3.28) nm; IR (KBr) *ν*_max_ 3412, 2937, 1676, 1450, 1290, 1206, 1140, 1030, 842, 801, 762, 723, 701, 520 cm^−1^; ^1^H-NMR (D_2_O, 400 MHz) and ^13^C-NMR (D_2_O, 100 MHz); see Table 1 for data. High-resolution electrospray ionization-mass spectrometry (HR-ESI-MS) gave a prominent quasi-molecular ion peak at *m*/*z* 587.23765 [M + H]^+^ (Figure S5), corresponding to the molecular formula C_25_H_38_N_4_O_10_S (calcd. for C_25_H_39_N_4_O_10_S, 587.23814), with nine degrees of unsaturation. The UV spectrum showed maximal absorptions at 216, 253, and 291 nm, which was similar to those of ustiloxins A (**2**), B (**3**), and C [6,7,8], suggesting the presence of a similar skeleton in **1**. The IR spectrum of **1** showed absorption bands for hydroxyl (3412 cm^−1^), and carbonyl (1676 cm^−1^) (Figure S6). The ^13^C-NMR (Table 1, Figure S7) and HMQC spectra (Figure S8) of **1** revealed the presence of four carbonyls (*δ*_C_ 175.9, 170.7, 170.0, 165.7), six sp^2^-hybridized carbons that included four quaternary carbons (*δ*_C_ 151.8, 145.6, 136.1, 127.7) and two methines (*δ*_C_ 113.6, 123.8), one oxygenated quaternary carbon (*δ*_C_ 86.8), five sp^3^-hybridized methines (*δ*_C_ 73.5, 66.2, 59.7, 59.2, 28.4), four sp^3^-hybridized methylenes (*δ*_C_ 60.1, 55.4, 43.6, 31.9), and five methyls (*δ*_C_ 31.7, 20.8, 18.0, 17.6, 7.5). The ^1^H-NMR spectrum (Table 1, Figure S9) of **1** was similar to that of ustiloxin C (Figure 1) [6], except that an isopropyl group (*δ*_H_ 0.87 (3H, d), 0.77 (3H, d), and 1.88 m) in **1** replaced that of a methyl group in ustiloxin C. This was confirmed by analysis of HMBC spectrum, in which correlations from H_3_-25 (*δ*_H_ 0.77, d) and H_3_-26 (*δ*_H_ 0.87, d) to C-24 (*δ*_C_ 28.4, CH) and C-6 (*δ*_C_ 59.7, CH) were discerned (Figure 2 and Figure S10). On the basis of their common biosynthetic origin, the absolute configuration of **1** was assumed to be the same as those of the co-isolated ustiloxins (Figure 1). Thus, the structure of **1** was determined, and named ustiloxin G.

### 2.2. Cytotoxicity and Phytotoxicity Assays

**Figure 3:**
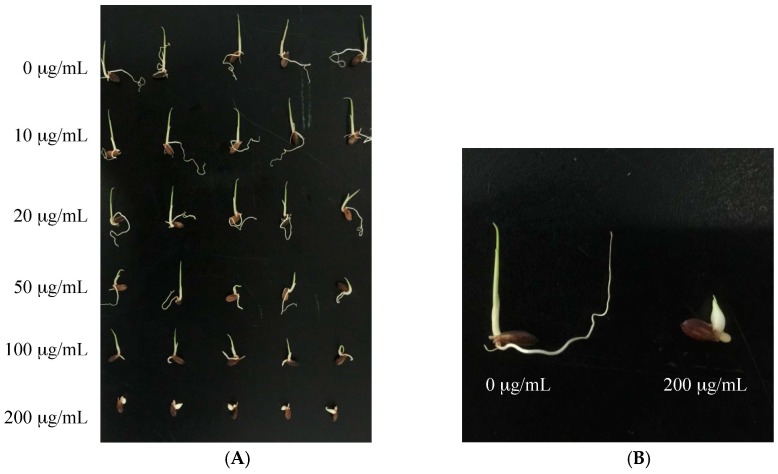
Inhibitory activity of ustiloxin G (**1**) on the radicle and germ elongation of the seeds of rice variety Lijiang. (**A**) is the photo of the rice seeds treated with ustiloxin G (**1**) at concentrations of 0, 10, 20, 50, 100, and 200 µg/mL from top to bottom; (**B**) is the amplified photo of the rice seeds treated with 0 µg/mL (the negative control) and 200 µg/mL of ustiloxin G (**1**) to show the swelling of the root and germs.

Compounds **1**–**5** were evaluated for their cytotoxic and phytotoxic activities (Table 2 and Table 3). Ustiloxin A (**2**) showed moderate activity against human tumor cell lines BGC-823 and A549, with IC_50_ values of 2.66 and 3.12 µM, respectively, while ustiloxin B (**3**) showed activity against BGC-823, HCT116, NCI-H1650, and HepG2 cells with IC_50_ values of 1.03, 7.2, 21.6, and 13.0 µM, respectively. Ustiloxin G (**1**) showed weak activity against cell lines A549 and A375 with IC_50_ values of 36.5 and 22.5 µM, respectively (Table 2). Meanwhile, ustiloxins A (**2**), B (**3**), and G (**1**) showed strong inhibitory activities on seed radicle and germ elongation of two rice varieties, Lijiang and Zhonghua 11 (Table 3). At the concentration of 200 µg/mL, inhibition ratios of ustiloxins A (**2**), B (**3**), and G (**1**) were at least 90% on radicle elongation, and 50% on germ elongation, the same effective as that of positive control (glyphosate). Ustiloxins A (**2**), B (**3**), and G (**1**) also induced abnormal swelling of the root and germs of rice seedlings (Figure 3B). The photos of the seeds of rice variety Lijiang treated with ustiloxin G (**1**) are shown in Figure 3. The results suggest that a thionyl structure in the side chain of C-12 should contribute to the inhibitory activity of ustiloxins A (**2**), B (**3**), and G (**1**) which was stronger than that of ustiloxins D (**4**) and F (**5**).

## 3. Conclusions

In conclusion, a new cyclopeptide, namely ustiloxin G (**1**), along with four known congeners ustiloxins A (**2**), B (**3**), D (**4**) and F (**5**) have been identified from rice FSBs. The structures of the compounds were elucidated based on 1D, 2D NMR and HR-ESI-MS analysis. Meanwhile, their cytotoxicity on human cancer cell lines and phytotoxicity on rice seed radicle and germ elongation were evaluated. Ustiloxins A, B, and G, each with a thionyl group in the side chain, showed higher toxicity than those without (i.e., ustiloxins D and F), which is consistent with the previous report [6]. Five ustiloxins (ustiloxins A, B, C, D, and F) have been isolated and identified from rice FSBs previously [6,7]. In this study, ustiloxin C was not isolated, possibly because of its very low content [19]. Instead, we identified a new cyclopeptide, ustiloxin G (**1**), which is an analogue of ustiloxin C. Other minor ustiloxins in rice FSBs should be further identified in detail. Ustiloxins have been considered as a kind of mycotoxin in rice FSBs and false smut pathogen-contaminated rice food and forage. They have created concerns for food and feed safety. Control of ustiloxin pollution and production, as well as studies for their biosynthesis, action mechanisms, and potential applications should be paid more attention in the future.

## 4. Experimental Section

### 4.1. General Experimental Procedures

Optical rotations were recorded on a Rudolph Autopol IV automatic polarimeter (Rudolph Research Analytical, Hackettstown, NJ, USA). UV spectra were recorded on a TU-1810 UV–vis spectrophotometer (Beijing Persee General Instrument Co., Ltd., Beijing, China). Infrared (IR) spectra were measured on a Thermo Nicolet Nexus 470 FT-IR spectrometer (Thermo Electron Scientific Instrument Crop., Fitchburg, WI, USA). High-resolution electrospray ionization mass spectrometry (HR-ESI-MS) spectra were recorded on a Bruker Apex IV FTMS instrument (Bruker Daltonics, Bremen, Germany) for ustiloxin G (**1**), and an LC 1260-Q-TOF/MS 6520 instrument (Agilent Technologies, Santa Clara, CA, USA) for ustiloxins D (**4**) and F (**5**). ^1^H-, ^13^C-, and 2D NMR (HMQC, HMBC) spectra were measured on an Avance 400 NMR spectrometer (Bruker BioSpin, Zürich, Switzerland). Chemical shifts were expressed in ppm as *δ* values relative to tetramethylsilane (TMS) as an internal standard, and coupling constants (*J*) are in hertz.

The high-performance liquid chromatography (HPLC) analysis was performed using a Shimadzu Prominence LC-20A instrument with an SPD-M20A photodiode array detector (Shimadzu, Kyoto, Japan) and an analytical reversed-phase Luna C18 column (250 mm × 4.6 mm, 5 μm; Phenomenex, Torrance, CA, USA). Semi-preparative HPLC separation was carried out on a Lumtech instrument (Lumiere Tech. Ltd., Beijing, China) equipped with a K-501 pump (flow rate: 3 mL/min) and a K-2501 UV detector (Knauer, Berlin, Germany) using a Luna reversed-phase C18 column (250 mm × 10 mm, 5 μm, Phenomenex, Torrance, CA, USA). Precoated silica gel GF-254 plates (Qingdao Marine Chemical Company, Qingdao, China) were used for analytical thin-layer chromatography (TLC). Spots were visualized under UV light (254 nm and 365 nm) or by spraying with 0.2% ninhydrin–acetone (w/v) reagent.

The macroporous adsorption resin SP207 was purchased from Mitsubishi Chemical Holdings, Japan. The GH gel ODS-AQ was purchased from Daiso, Japan. The Sephadex G-15 was purchased from GE Healthcare, USA. Methanol used for analytical and semi-preparative HPLC was chromatography grade and was purchased from Xilong Chemical Company (Shantou, China). Ultrapure water was used throughout the experiment. All other reagents were of analytical grade.

### 4.2. Materials

The rice FSBs were collected from Linyi (118.24° E, 35.15° N), the southwestern part of Shandong Province of China in October 2012. The materials were left to dry in shade at room temperature to a constant weight, and were then stored in sealed plastic bags at −20 °C until required.

### 4.3. Extraction and Isolation

After rice FSBs were extracted with the tested solvents (i.e., methanol, ethanol, acetone, and water), respectively, water was proved to be the most efficient, and selected for the extraction of ustiloxins from the materials. The dry rice FSBs (6.38 kg) were extracted with deionized water with the ratio of rice FSBs to water as 1:3 (kg/L) at room temperature five times (24 h for each time) and shaken vigorously occasionally. After filtration, the filtrates were combined and concentrated under vacuum at 60 °C by a rotary evaporator to give a water extract residue (2870 g). A new ustiloxin analogue in trace amounts was primarily speculated by analyzing the HPLC profile of the water extract of rice FSBs (Figure S11). The water extract was subjected to chromatography over macroporous adsorption resin SP207 eluting with a gradient of EtOH–H_2_O (0:100, 40:60, 100:0, *v*/*v*) to obtain three fractions (Frs. A~C). Fr. B (74 g) eluted with 40% aqueous ethanol containing the main ustiloxins were used for further separation. Fr. B was chromatographed on an ODS-AQ column (38 mm × 360 mm) eluting with 800 mL of MeOH:H_2_O containing 0.02% trifluoroacetic acid (TFA) (0:100, 0.3:100, 0.5:100, 1:100, 1:99, 3:97, 5:95, 7:93, 10:90, 15:85, 20:80, 30:70, 50:50, 100:0, *v*/*v*) respectively to yield 10 subfractions (Fr. B1~B10).

Fr. B2 (786 mg) was further subjected to gel permeation chromatography over Sephadex G-15, eluting with H_2_O (250 mL) to yield four subfractions (Fr. B2-1 to B2-4), among which Fr. B2-2 was a pure compound **3** (23 mg). Fr. B2-4 was purified by semi-preparative HPLC using MeOH–H_2_O (0.02% TFA) (15:85, *v*/*v*) as eluent to produce **5** (11 mg). Similarly, Fr. B4 (890 mg) was subjected size-exclusion chromatography over Sephadex G-15 eluting with H_2_O (250 mL) to yield **2** (48 mg). Fr. B5 (1.2 g) was further chromatographed on a Sephadex G-15 column with H_2_O (250 mL) as an eluent to obtain four subfractions (Fr. B5-1 to Fr. B5-4), among which Fr. B5-3 was further purified by semi-preparative HPLC eluting with MeOH–H_2_O (0.02% TFA) (20:80, *v*/*v*) to afford **4** (13 mg). Fr. B8 (3.8 g) was further chromatographed over an ODS-AQ column eluting with MeOH:H_2_O (containing 0.02% TFA) (0:100, 5:95, 10:90, 15:85, 20:80, 30:70, 50:50, 100:0, *v*/*v*) to obtain nine subfractions (Fr. B8-1 to Fr. B8-9), among which Fr. B8-4 was further purified by Sephadex G-15 column to afford **1** (8 mg).

### 4.4. Cytotoxic Assay

Cytotoxicity of the compounds were tested against human carcinoma cells using the microculture methyl-thiazolyl-tetrazolium (MTT) assay as described previously [20]. Briefly, the cell lines were maintained in a humidified atmosphere containing 5% CO_2_ at 37 °C. For preparing working solution, 1 μmol of each purified ustiloxin was dissolved in 10 mL of ultrapure water to obtain the stock solution (100 μM), which was further diluted to a series of concentrations (50, 25, 12.5, 6.25, 3.125, 1, 0.1, 1 × 10^−2^, 1 × 10^−3^, 1 × 10^−4^, 1 × 10^−5^ μM) with ultrapure water. Normally, 200 μL of the cell suspensions (5 × 10^4^ cells/mL) was plated in 96-well culture plates at 37 °C. After 12 h, 20 μL of working solution was added to each well respectively and cultured at 37 °C for 24 h. Then, MTT was dissolved in saline to 5 mg/mL, and 20 μL of this solution was added to each well. After 4 h, the medium was removed, and then 100 μL of DMSO was added to dissolve any formazan crystals formed. Absorbance was then determined at 570 nm. The cell lines of colon cancer cells (HCT-116), non-small-cell lung carcinoma cells (NCI-H1650), gastric cancer cells (BGC-823), and liver hepatocellular carcinoma cells (HepG2) were tested for all isolated compounds. In addition, lung adenocarcinoma cells (A549) were tested on ustiloxins A (**2**), B (**3**), and G (**1**), and melanoma cells (A375) were tested on ustiloxin G (**1**). Ultrapure water and taxol were used as the negative and positive controls, respectively. All experiments were performed with three replicates.

### 4.5. Inhibitory Activity Assay on the Radicle and Germ Elongation of Rice Seeds

Phytotoxicity of the compounds was evaluated by their inhibitory activities on the radicle and germ elongation of rice (*Oryza sativa*) seeds. Two varieties (Lijiang and Zhonghua 11) of the seeds, which were obtained from Prof. Zejian Guo (Department of Plant Pathology and collected in 2015, China Agricultural University) were used.

The bioassay was performed using the method described previously [12] with some modifications. Briefly, five 3-day germinated rice seeds of each rice variety were sown onto each well of a 24-well plate, containing 200 μL of working solution. For preparing working solution, 1 mg of each purified compound was dissolved in 1 mL of ultrapure water to obtain the stock solution (1 mg/mL), which was further diluted to a series of concentrations (200, 100, 50, 20, and 10 μg/mL) with ultrapure water. The ultrapure water was used as the negative control, and glyphosate (*N*-(phosphonomethyl)glycine) at the same concentrations as the positive control. Plates were incubated in a moist chamber at 25 °C in the dark. The length of each radicle or germ was measured after treatment for 48 h. The inhibition of radicle or germ elongation was calculated as follows: Inhibition (%) = [(*L*_c_ − *L*_t_)/*L*_c_] × 100, where *L*_c_ is the radicle or germ length of the control group, and *L*_t_ is the length of the treated group.

All experiments were performed with three replicates, and the results were represented by their mean values and the standard deviations (SD). The data were carried out using analysis of variance (one-way ANOVA) to detect significant differences by PROC ANOVA of SAS version 8.2. Different letters indicated that the data were significantly different at *p* ≤ 0.05.

## Figures and Tables

**Table 1:** ^1^H-NMR and ^13^C-NMR data of ustiloxin G (**1**) in D_2_O (*δ* in ppm, *J* in Hz).

Position	*δ*_C_ *^a^*	*δ*_H_, mult. (*J* in Hz) *^b^*	HMBC *^c^*
C (2)	86.8	-	-
CH (3)	59.2	4.83, s	C (2), C (17), CH_3_ (21)
C (5)	170.7	-	-
CH (6)	59.7	4.13, d (10.1)	C (5), C (8), CH (24)
C (8)	165.7	-	-
CH (9)	66.2	4.29, d (9.9)	C (8), C (10)
NCH_3_ (9)	31.7	2.77, s	C (9)
CH (10)	73.5	4.93, d (10.1)	C (9), C (11), C (12)
C (11)	127.7	-	-
C (12)	136.1	-	-
CH (13)	113.6	7.58, s	C (11), C (12), C (14), C (15)
C (14)	151.8	-	-
C (15)	145.6	-	-
CH (16)	123.8	7.08, s	-
C (17)	170.0	-	-
CH_2_ (19)	43.6 *^d^*	3.77, s	C (17), COOH (20)
COOH (20)	175.9 *^e^*	-	-
CH_3_ (21)	20.8	1.75, s	C (2), CH (3), CH_2_ (22)
CH_2_ (22)	31.9 *^e^*	1.70, m; 2.21, m	-
CH_3_ (23)	7.5	1.08, t (7.3)	-
CH (24)	28.4	1.88, m	-
CH_3_ (25)	18.0	0.77, d (6.7)	CH (6), CH (24), CH_3_ (26)
CH_3_ (26)	17.6	0.87, d (6.6)	CH (6), CH (24), CH_3_ (25)
CH_2_ (2’)	60.1	2.98, br.d (13.6); 3.41, m	C (12)
CH_2_ (3’)	55.4	4.04, m	-

Note: *^a^* Recorded at 100 MHz. *^b^* Recorded at 400 MHz. *^c^* HMBC correlations are from proton(s) stated to the indicated carbon. *^d^* ^13^C shifts are assigned by HMQC experiments. *^e^* ^13^C shifts are assigned by HMBC experiments.

**Table 2:** Cytotoxicity of ustiloxins (**1**–**5**) on human tumor cell lines.

Compound	IC_50_ (µM)					
	HCT116	NCI-H1650	BGC-823	HepG2	A549	A375
Ustiloxin G (**1**)	>50	>50	>50	>50	36.5	22.5
Ustiloxin A (**2**)	>50	>50	2.66	>50	3.12	-
Ustiloxin B (**3**)	7.2	21.6	1.03	13.0	>50	-
Ustiloxin D (**4**)	>50	>50	>50	>50	-	-
Ustiloxin F (**5**)	>50	>50	>50	>50	-	-
Taxol (CK^+^)	1.9 × 10^−3^	1.1	1.07 × 10^−4^	1.46 × 10^−2^	2.3 × 10^−2^	2.2 × 10^−2^

Note: HCT-116, colon cancer cell lines; NCI-H1650, non-small-cell lung carcinoma cells; BGC-823, gastric cancer cells; HepG2, liver hepatocellular carcinoma cells; A549, lung adenocarcinoma cells; A375, melanoma cells; -, not tested. CK^+^ represents positive control.

**Table 3:** Inhibitory activity of the isolated ustiloxins (**1**–**5**) on the radicle and germ elongation of rice seeds.

Compound	Concentration (µg/mL)	Inhibition Ratio (%)			
		Seed Radicle		Seed Germ	
		Var. Zhonghua 11	Var. Lijiang	Var. Zhonghua 11	Var. Lijiang
Ustiloxin G (**1**)	10	27.27 ± 1.11 ghi	12.56 ± 1.68 jk	14.11 ± 4.51 mn	2.74 ± 4.76 l
	20	31.67 ± 5.85 ef	17.00 ± 5.12 ij	14.68 ± 5.69 mn	8.89 ± 4.13 kl
	50	54.14 ± 4.20 d	37.64 ± 1.21 g	36.29 ± 4.55 efgh	27.96 ± 3.32 f
	100	90.16 ± 3.62 a	90.33 ± 1.33 ab	39.33 ± 6.57 defg	36.95 ± 5.23 e
	200	93.40 ± 1.91 a	95.19 ± 1.92 a	50.45 ± 5.49 abc	65.01 ± 3.57 abc
Ustiloxin A (**2**)	10	12.27 ± 6.71 l	17.83 ± 2.56 ij	13.25 ± 4.37 mn	11.38 ± 5.51 jk
	20	56.16 ± 2.30 d	43.57 ± 0.61 f	26.59 ± 4.37 ijkl	18.00 ± 2.33 ghij
	50	89.14 ± 4.38 a	87.38 ± 0.79 b	42.04 ± 3.50 cdef	39.01 ± 6.84 e
	100	93.72 ± 0.54 a	94.53 ± 0.36 a	45.47 ± 4.33 bcd	50.59 ± 6.36 d
	200	95.29 ± 1.63 a	96.64 ± 0.36 a	52.33 ± 4.37 ab	60.27 ± 3.06 bc
Ustiloxin B (**3**)	10	11.90 ± 6.45 l	4.16 ± 0.66 l	3.30 ± 1.37 op	13.54 ± 2.25 hijk
	20	20.22 ± 3.98 ijk	14.27 ± 0.83 jk	12.02 ± 6.29 mno	20.63 ± 3.20 fgh
	50	54.78 ± 3.09 d	53.24 ± 4.98 e	35.60 ± 3.96 efghi	37.98 ± 3.83 e
	100	87.80 ± 5.14 ab	86.62 ± 1.98 b	44.72 ± 5.85 bcde	49.65 ± 2.13 d
	200	93.04 ± 2.10 a	95.32 ± 1.32 a	55.61 ± 2.75 a	64.39 ± 2.13 abc
Ustiloxin D (**4**)	10	13.55 ± 4.17 kl	4.43 ± 5.47 l	10.98 ± 4.03 no	10.73 ± 3.81 jk
	20	23.31 ± 7.38 hij	5.42 ± 3.45 l	12.28 ± 5.15 mno	11.67 ± 5.89 ijk
	50	41.47 ± 4.88 e	11.89 ± 3.95 jk	16.19 ± 3.07 mn	11.87 ± 7.91 ijk
	100	90.79 ± 0.94 a	79.92 ± 6.26 c	25.48 ± 7.06 jkl	19.92 ± 5.40 fghi
	200	93.90 ± 2.11 a	94.92 ± 0.79 a	31.86 ± 5.68 ghij	38.76 ± 5.68 e
Ustiloxin F (**5**)	10	0.75 ± 6.44 ml	5.07 ± 4.95 l	1.89 ± 4.86 p	7.07 ± 6.85 kl
	20	9.59 ± 3.80 l	8.97 ± 2.96 jk	9.36 ± 1.62 nop	7.90 ± 4.3 k1
	50	16.51 ± 3.45 jkl	22.17 ± 2.48 hi	14.97 ± 5.84 mn	9.14 ± 4.97 kl
	100	23.78 ± 4.24 hij	26.72 ± 5.84 h	17.77 ± 6.85 lmn	15.16 ± 6.71 hijk
	200	36.83 ± 2.82 ef	38.25 ± 6.05 fg	18.71 ± 4.86 lmn	24.57 ± 3.42 fg
Glyphosate (CK^+^)	10	28.24 ± 4.90 gh	36.84 ± 2.91 g	21.16 ± 6.15 klm	20.99 ± 2.92 fgh
	20	41.52 ± 5.20 e	62.91 ± 5.03 d	29.21 ± 5.23 hijk	37.82 ± 4.05 e
	50	68.88 ± 3.85 c	85.08 ± 3.80 bc	35.36 ± 6.97 fghi	59.32 ± 5.06 c
	100	81.05 ± 2.04 b	93.64 ± 2.66 a	57.60 ± 3.44 a	68.21 ± 0.81 ab
	200	87.72 ± 1.10 ab	95.61 ± 2.74 a	58.93 ± 6.04 a	71.36 ± 2.68 a

Note: Each value represents the means of triplicate ± standard deviations. Different letters indicate significant differences among treatments in each column including different compounds and their concentrations at *p* ≤ 0.05. CK^+^ represents positive control.

## Supplementary Materials

The following are available online at www.mdpi.com/2072-6651/9/2/54/s1. Figure S1. ^1^H-NMR spectrum of ustiloxin D (**4**) (CD_3_OD, 400 MHz); Figure S2. ^13^C-NMR spectrum of ustiloxin D (**4**) (CD_3_OD, 100 MHz); Figure S3. ^1^H-NMR spectrum of ustiloxin F (**5**) (CD3OD, 400 MHz); Figure S4. ^13^C-NMR spectrum of ustiloxin F (**5**) (CD_3_OD, 100 MHz); Figure S5. HR-ESI-MS spectrum of ustiloxin G (**1**); Figure S6. IR spectrum of ustiloxin G (**1**); Figure S7. ^13^C-NMR spectrum of ustiloxin G (**1**) (D_2_O, 100 MHz); Figure S8. HMQC spectrum of ustiloxin G (1) (D_2_O); Figure S9. ^1^H-NMR spectrum of ustiloxin G (**1**) (D_2_O, 400 MHz); Figure S10. HMBC spectrum of ustiloxin G (**1**) (D_2_O); Figure S11. HPLC chromatogram of the water extract of rice FSBs. Ustiloxins A, B, D, F and G were abbreviated as UA, UB, UD, UF and UG, respectively. (A) HPLC profile of the water extract of rice FSSs; (B) The UV absorption spectrum of UG.
