# Supplementary material for: Ustiloxin G, a New Cyclopeptide Mycotoxin from Rice False Smut Balls
Source: Toxins (Basel). 2017 Feb 10;9(2):54. doi: 10.3390/toxins9020054 (PMC5331433; doi:10.3390/toxins9020054)
Supplement: Supplementary file 1 [file toxins-09-00054-s001.pdf]

Supplementary Materials: Ustiloxin G, a New Cyclopeptide Mycotoxin from Rice False Smut Balls

Xiaohan Wang, Jian Wang, Daowan Lai, Weixuan Wang, Jungui Dai, Ligang Zhou and Yang Liu

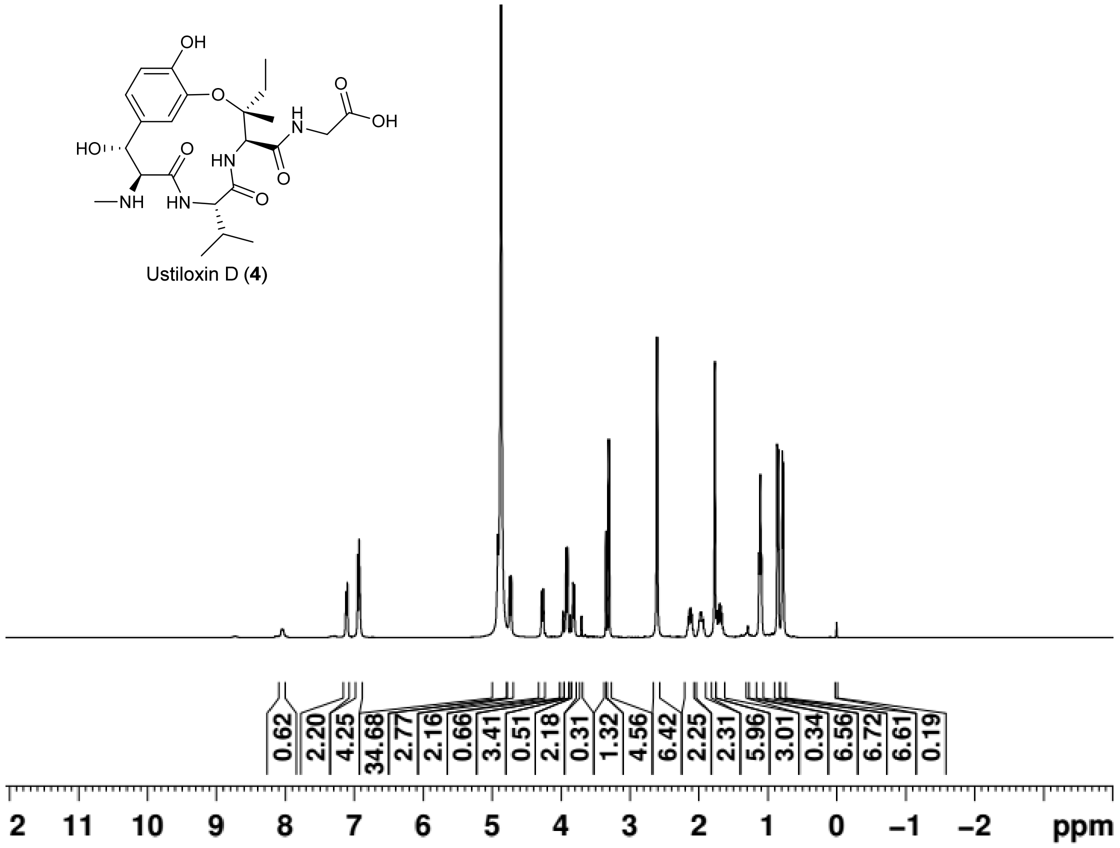

Figure S1. ^{1}H-NMR spectrum of ustiloxin D (**4**) (CD_3OD, 400 MHz).

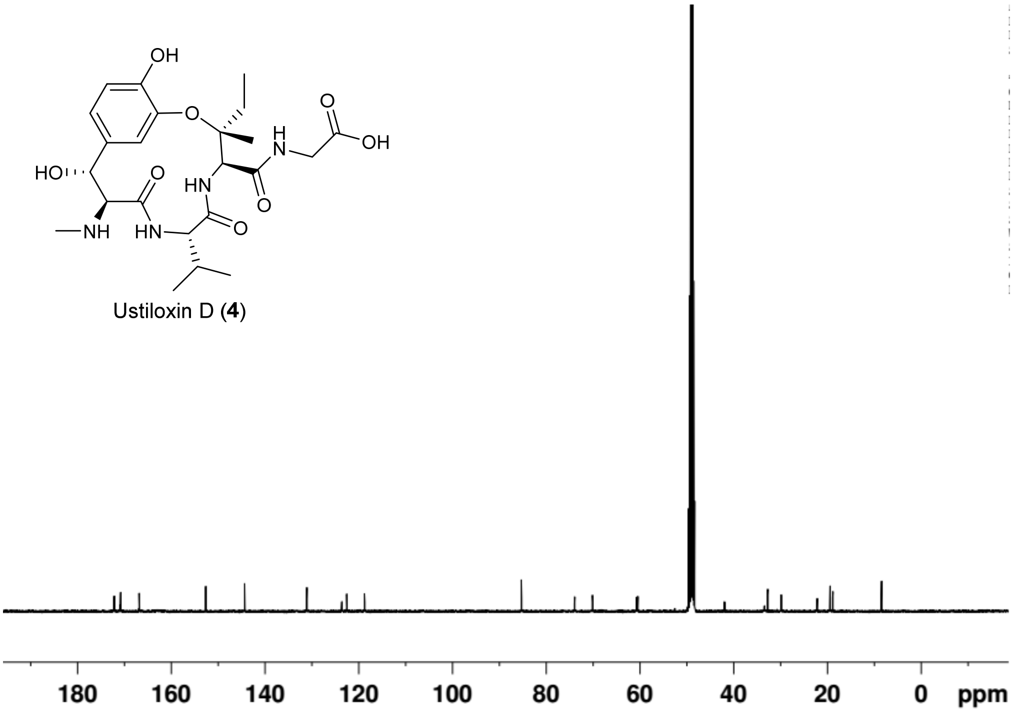

Figure S2. ^{13}C-NMR spectrum of ustiloxin D (**4**) (CD_3OD, 100 MHz).

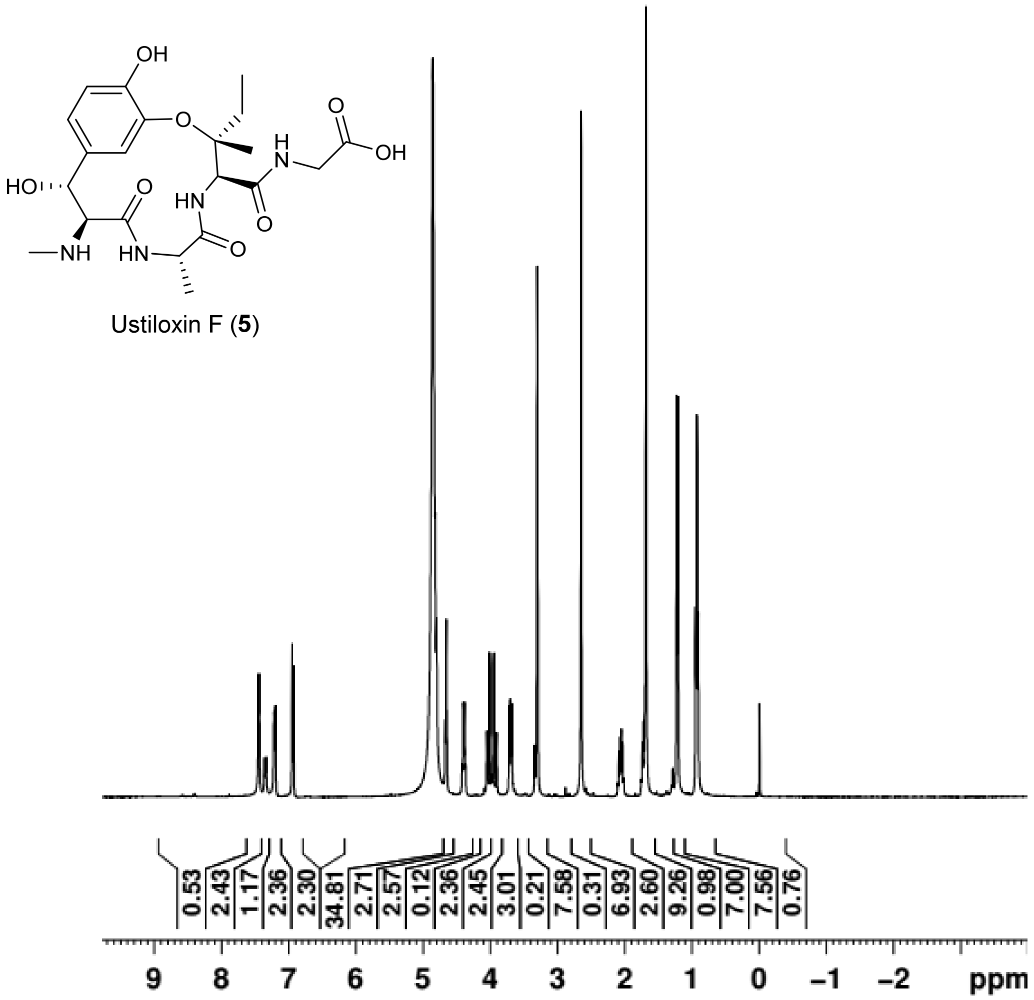

Figure S3. ^{1}H-NMR spectrum of ustiloxin F (**5**) (CD_3OD, 400 MHz).

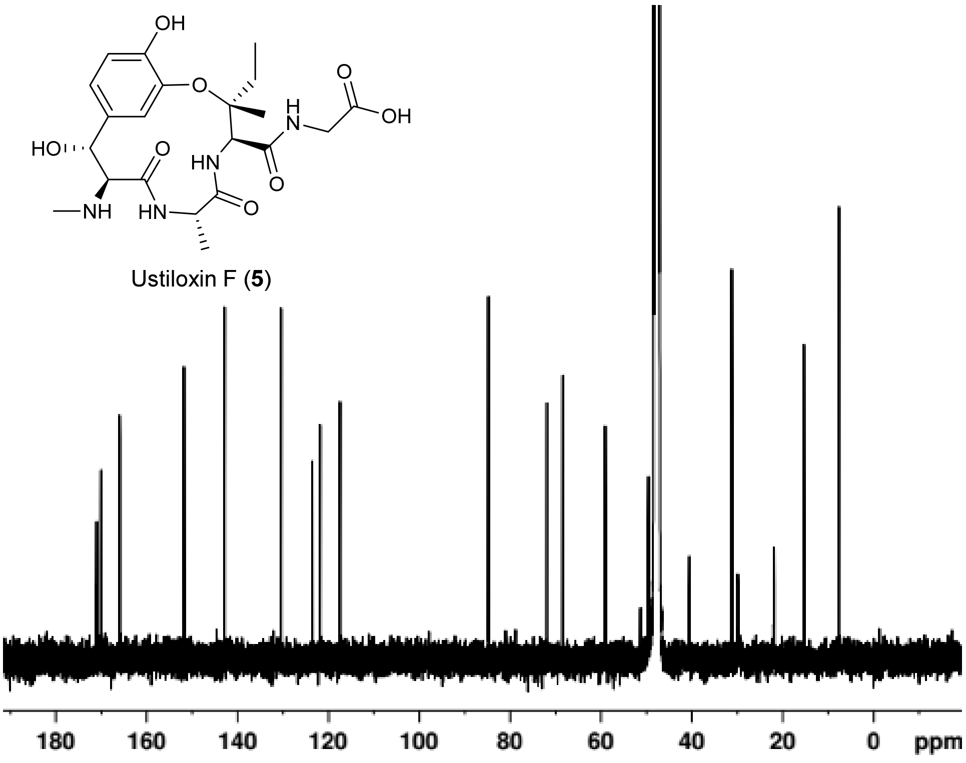

Figure S4. ^{13}C-NMR spectrum of ustiloxin F (**5**) (CD_3OD, 100 MHz).

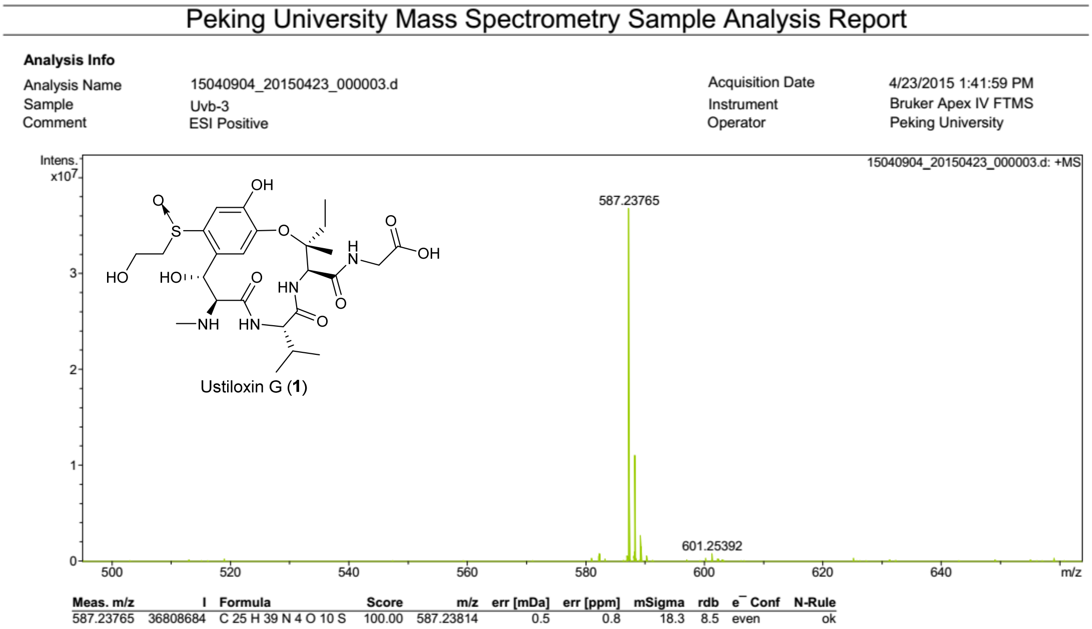

Figure S5. HR-ESI-MS spectrum of ustiloxin G (**1**).

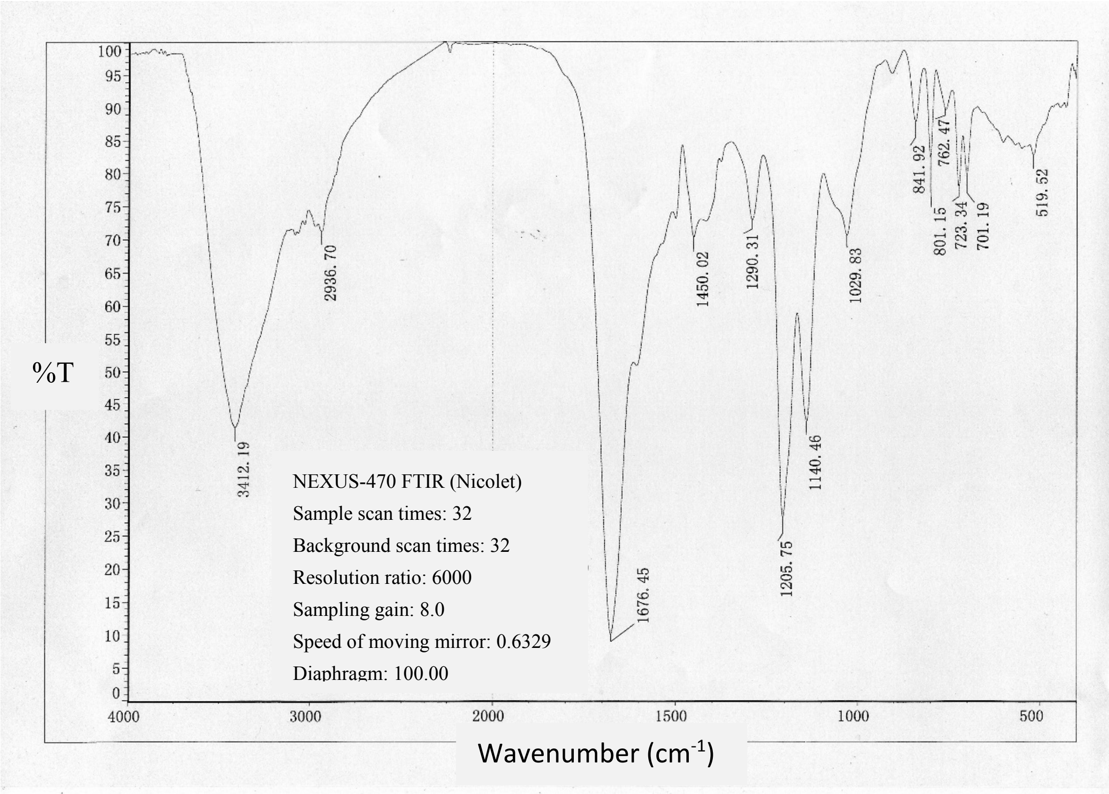

Figure S6. IR spectrum of ustiloxin G (**1**).

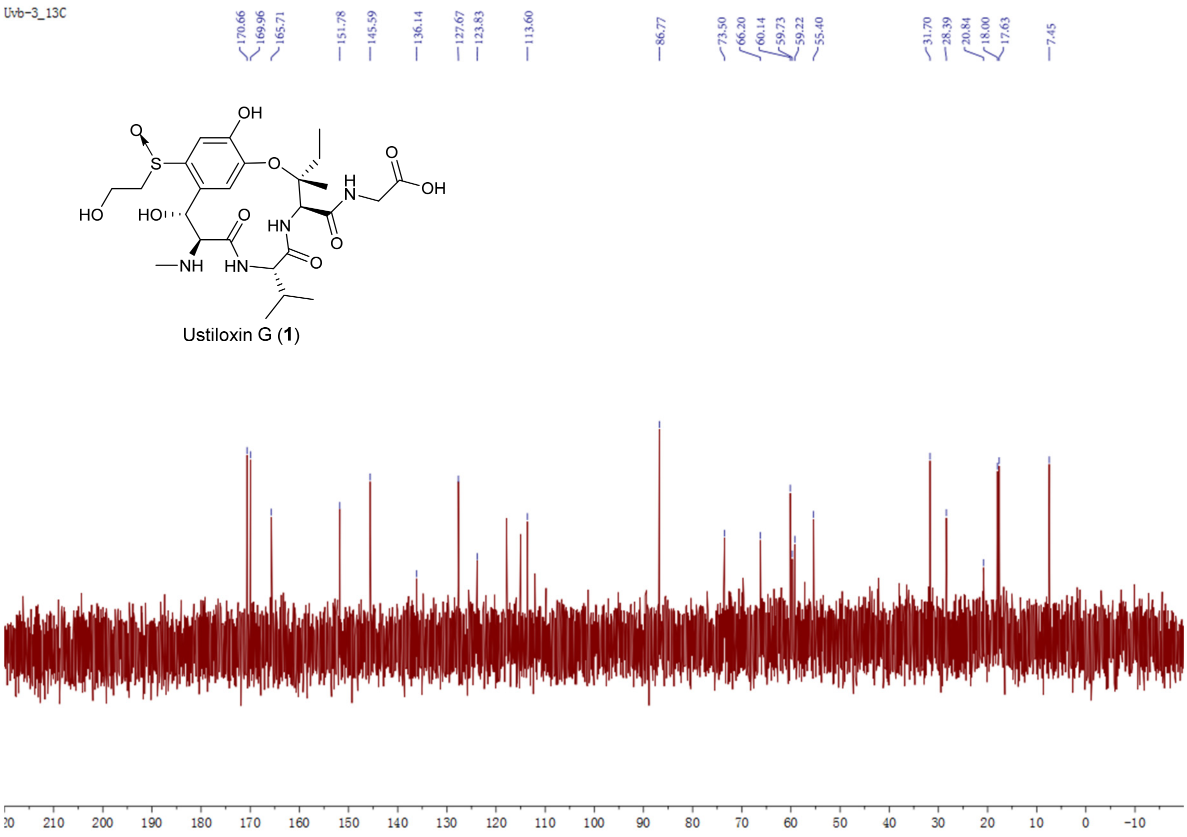

Figure S7. ^{13}C-NMR spectrum of ustiloxin G (**1**) (D_2O, 100 MHz).

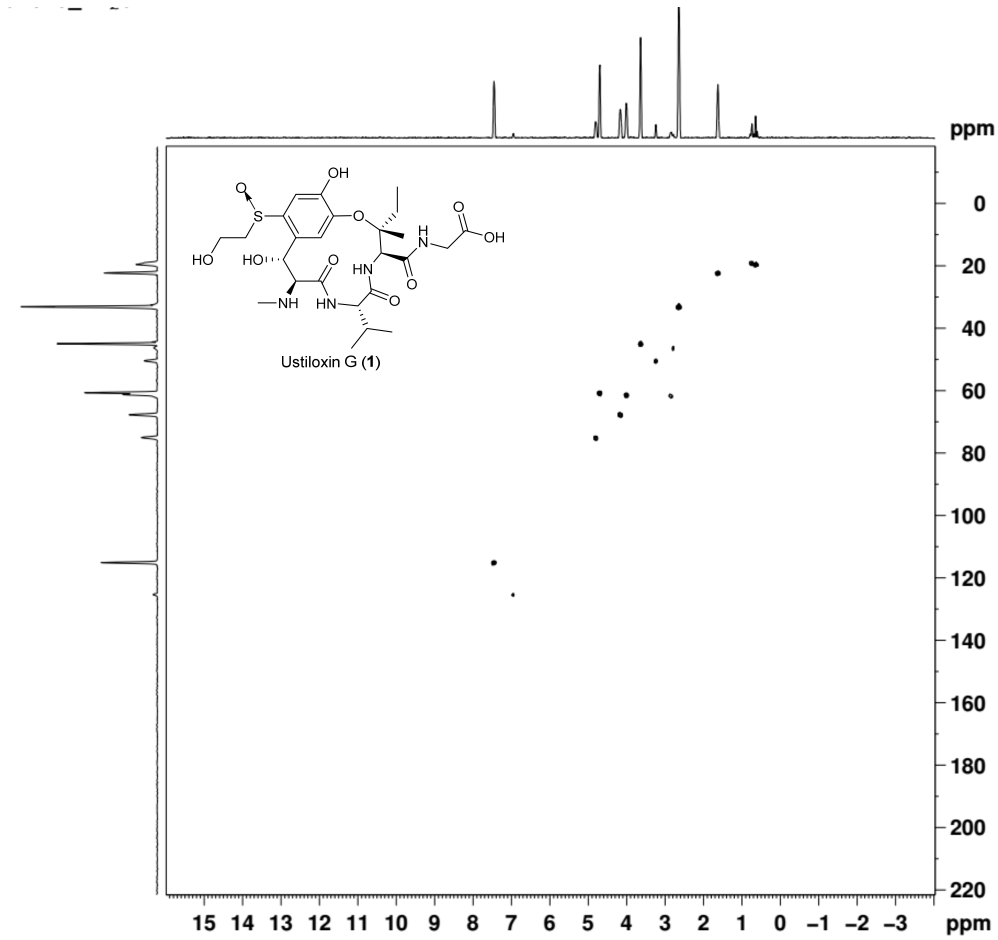

Figure S8. HMQC spectrum of ustiloxin G (**1**) (D_2O).

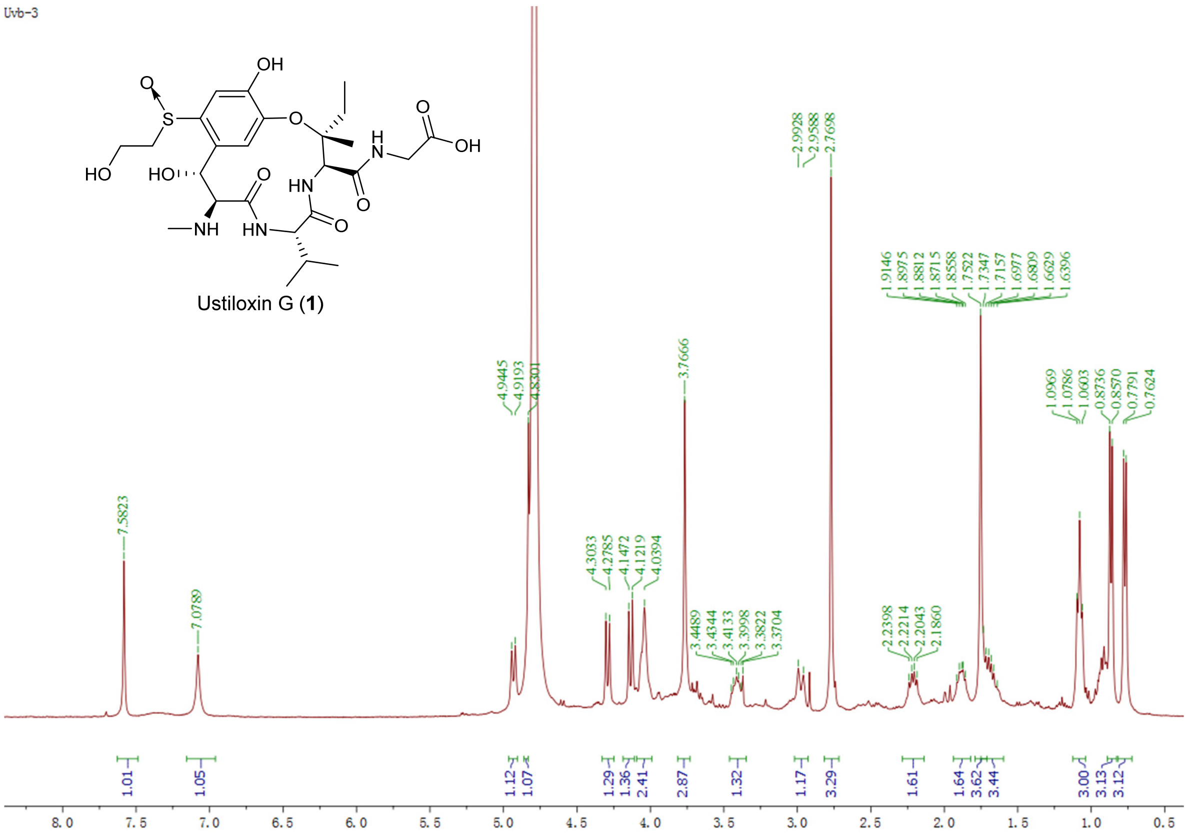

Figure S9. ^{1}H-NMR spectrum of ustiloxin G (**1**) (D_2O, 400 MHz).

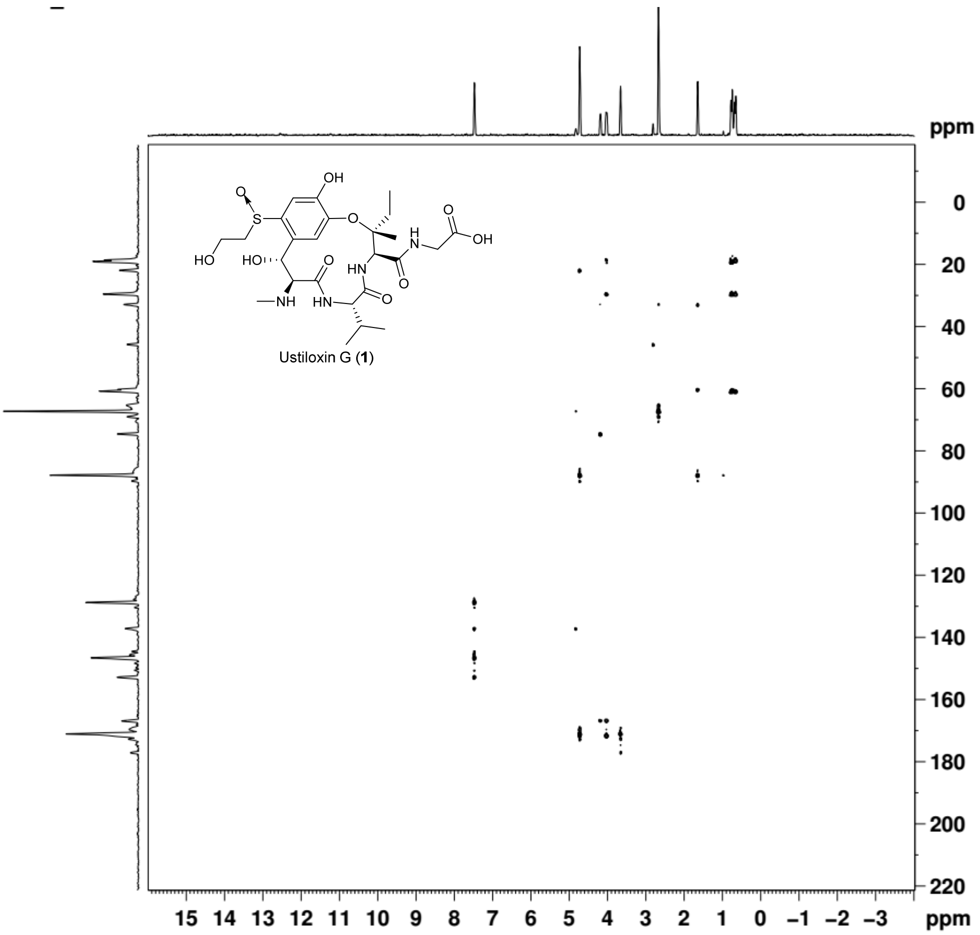

Figure S10. HMBC spectrum of ustiloxin G (**1**) (D_2O).

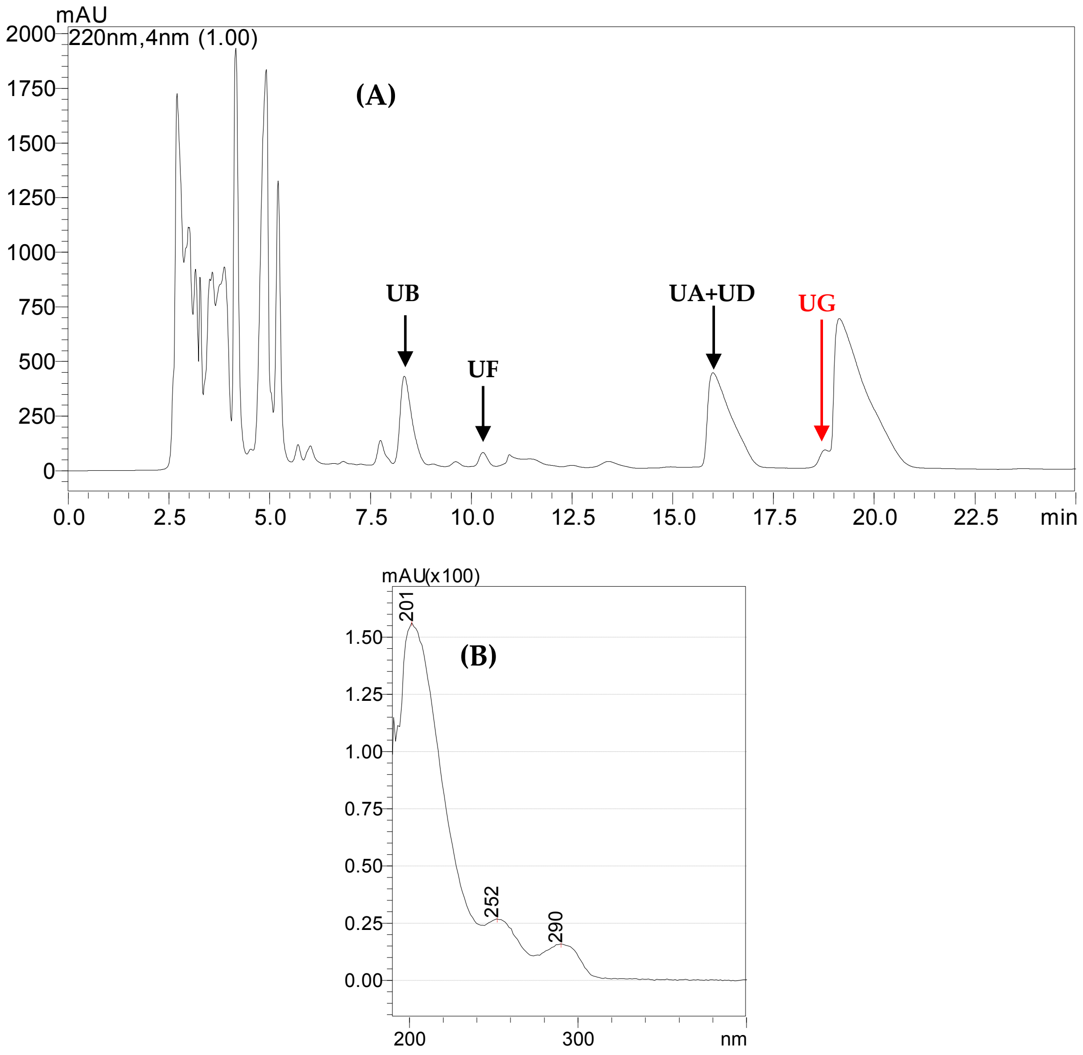

Figure S11. HPLC chromatogram of the water extract of rice FSBs. Ustiloxins A, B, D, F and G were abbreviated as UA, UB, UD, UF and UG, respectively. (**A**) HPLC profile of the water extract of rice FSSs; (**B**) The UV absorption spectrum of UG.
